# Peripherally inserted central venous catheter in upper extremities leads to an increase in D-dimer and deep vein thrombosis in lower extremities

**DOI:** 10.1186/s12959-021-00275-w

**Published:** 2021-04-09

**Authors:** Wanli Liu, Lianxiang He, Wenjing Zeng, Liqing Yue, Jie Wei, Shuangshuang Zeng, Xiang Wang, Zhicheng Gong

**Affiliations:** 1 Teaching and Research Section of Clinical Nursing, Xiangya Hospital, Central South University, Changsha, Hunan People’s Republic of China; 2 Institute for Rational and Safe Medication Practices, National Clinical Research Center for Geriatric Disorders, Xiangya Hospital, Central South University, Changsha, Hunan People’s Republic of China; 3 Department of Pharmacy, Xiangya Hospital, National Clinical Research Center for Geriatric Disorders, Central South University, Hunan 410008 Changsha, People’s Republic of China

**Keywords:** Peripherally inserted central venous catheter, Lower extremity deep venous thrombosis, D-dimer, Neurology department

## Abstract

**Background:**

The purpose of this study is to elucidate the association between peripherally inserted central venous catheter (PICC) in upper extremities and lower extremity deep venous thrombosis (LEDVT) by observing the changes in D-dimer.

**Methods:**

This was a retrospective cohort study with 3452 patients (104 inserted with PICCs and 3348 without PICC) enrolled at the neurology department from April 1, 2017 to April 1, 2020. The patients underwent color Doppler ultrasound (CDU) and D-dimer examinations. LEDVT-related factors and D-dimer value were analyzed before and after PICC insertion. The predictive value of D-dimer for LEDVT was also evaluated.

**Results:**

Univariate logistic regression analysis showed that PICC insertion increased the risk of LEDVT by 9 times and promoted the increase of D-dimer by 5 times. After risk adjustment, multivariate logistic regression analysis showed that PICC insertion increased the risk of LEDVT by 4 times and tripled the risk of D-dimer increase. The concentration of D-dimer was significantly increased after PICC insertion. D-dimer was unsuitable for excluding venous thrombosis in patients inserted with PICCs.

**Conclusions:**

PICC insertion increases the level of D-dimer and the risk of LEDVT. The risks of venous thrombosis need to be assessed in patients inserted with PICCs to ensure the expected clinical outcomes.

## Introduction

A peripherally inserted central venous catheter (PICC) is a tube that extends to the superior vena cava or inferior vena cava through superior limb basilic vein, median cubital vein, cephalic vein, brachial vein or external jugular vein. A PICC can enter the body of a newborn at great saphenous vein of lower limb, temporal vein of head or retroauricular vein. The use of PICC can reduce the pain of repeated puncture and the incidence of drug extravasation, which is welcomed by clinical medical staff, patients and family members [1]. However, PICC insertion and displacement may damage the vascular intima [2]. PICC insertion-induced endothelial injury and stasis of blood flow coupled with medication-induced hypercoagulability constitute the Virchow’s triad for thrombosis [3]. Deep venous thrombosis (DVT) is a venous thromboembolic disorder defined as “the formation of a blood clot within a deep vein” [4]. The incidence of PICC-related symptomatic upper extremity DVT (UEDVT) varies between 6 and 18 %, but it can reach 25 % in a few circumstances such as malignancies [5, 6]. The prevalence of asymptomatic thrombosis ranges from 35 to 71.9 % [7–11].

PICC-related venous thrombosis is mainly mural thrombus and limited to the venous route where the catheter is located [12–14]. Increasing evidence has suggested that PICC insertion also increases the incidence of lower extremity DVT (LEDVT) and that PICC-related venous thrombosis can even exceed the range of infusion route [2, 3, 15, 16]. However, there is no explanation for this clinical phenomenon.

D-dimer is a specific degradation product of cross-linked fibrin. An increase in D-dimer reflects the enhancement of coagulation and fibrinolysis system, which can be used as a sensitive indicator of hypercoagulability [12, 17]. D-dimer is augmented in catheter-related venous thrombosis [18, 19], and the normal value of D-dimer can be used to exclude venous thrombosis [18, 20–23]. Nonetheless, it is not clear whether D-dimer is the medium or by-product of catheter-related venous thrombosis [15]. In this study, the association between PICC in upper extremities and LEDVT was investigated by observing the changes in D-dimer.

## Materials and methods

### Patients and study design

The study was approved by the ethics committee of Xiangya Hospital of Central South University (202,004,327) and conducted according to the Helsinki Declaration of Ethical Principles for Medical Research Involving Human Subjects.

This retrospective case cohort study involved 3452 patients who received color Doppler ultrasound (CDU) in the neurology department of Xiangya Hospital of Central South University, Hunan Province from April 1, 2017 to April 1, 2020. The clinical characteristics of the patients are listed in Table 1.

**Table 1:** Clinical characteristics of all patients

Factors	PICC	NO PICC
	*n* = 104	*n* = 3348
Age (year, mean ± standard deviation)	62.0 ± 14.0	61.5 ± 13.6
Male	61 (58.7 %)	2036 (60.8 %)
Malignant tumor	7 (6.7 %)	120 (3.6 %)
Recent surgery	18 (17.3 %)	222 (6.6 %)
Cerebral hemorrhage	30 (28.8 %)	206 (6.2 %)
Ischemic stroke	32 (30.8 %)	1581 (47.2 %)
Parkinson’s disease	2 (1.9 %)	228 (6.8 %)
Infection	6 (5.8 %)	331 (9.9 %)
BMI ≥ 25	9 (8.6 %)	22 (0.7 %)
Unconsciousness	80 (76.9 %)	847 (25.3 %)
Critical illness	11 (10.6 %)	163 (4.9 %)

Note: *PICC* refers to peripherally inserted central venous catheter; *BMI* refers to body mass index; malignant tumor refers to cases treated within the prior six months; recent surgery refers to neurosurgery (over two hours) performed within six months; critical illness refers to the circumstance where patients have a high risk of disease variation or death

The inclusion criteria of research objects were as follows: aged ≥ 18 years, admitted to the neurology department and receiving CDU from April 1, 2017 to April 1, 2020. The exclusion criteria were: (1) under 18 years old; (2) pregnant; (3) inserted with a PICC in the lower extremity; (4) diagnosed with venous thromboembolism within six months before admission. All the 3452 patients were indexed and given a Caprini score and routine D-dimer test at admission to the neurology department. Patients at high risk of venous thrombosis underwent CDU at admission. PICCs were used for long-term infusion in patients having poor vascular conditions such as unclear superficial veins of upper extremities and difficulty in peripheral short catheter insertion. Only 104 of the 3452 patients were inserted with PICCs.

CDU was performed at admission on unconscious patients or on those with a high-risk score or suspected symptoms of DVT such as swelling, pain, tenderness and pyrexia of lower extremities. During hospitalization, CDU was performed on patients when they showed thrombotic symptoms such as pain and swelling of lower extremities or on long-term bedridden patients. All the 3452 patients underwent CDU, among which 1360 patients received D-dimer test within seven days before the CDU examination. Only 101 of the 1360 patients were inserted with PICCs.

In summary, all patients received blood vessel assessment at admission/before infusion for deciding the use of PICC. PICC insertion was required when patients had poor vascular conditions such as unclear superficial veins of upper extremities and difficulty in peripheral short catheter insertion. CDU was performed within 24 h on patients showing swelling, redness, pain, fever and other thrombotic symptoms during the catheterization. Comatose or bedridden patients without thrombotic symptoms underwent general ultrasound examination by nurses regularly once a week during the catheterization; CDU was further performed if the assessment result indicated suspected venous thrombosis.

### D-dimer test

D-dimer test was performed within seven days after catheterization. Blood samples for D-dimer test were obtained before CDU examination. The blood was drawn from the antecubital vein with a clean 22-gauge butterfly needle to a 3-ml plastic tube containing 0.3 ml of 0.106 M trisodium citrate. The tube was gently inverted to mix the blood with trisodium citrate for three to six times. The whole blood was centrifuged at 2000 ×g for 20 min at 20 °C. The automated and rapid STA Liatest® D-dimer assay was used for measurement of D-dimer. The STA Liatest® assay has high sensitivity (median value ≥ 95 %) which meets the requirements of the Food and Drug Administration and can be used to rule out venous thrombosis [24, 25]. The cut-off value for negative DVT was 0.5 mg/L. The detection methods or reagents adopted by the blood laboratory of the hospital remained unchanged during the research period.

### CDU

CDU was performed within seven days after D-dimer test by an experienced CDU technician using Philips Epiq 5 and a high-resolution linear array transducer (9–13 MHz). The criteria for the diagnosis of venous thrombosis were as follows [26–28]: the lumen cannot be compressed despite firm compression with the transducer probe; defective blood flow signal in the lumen; solid return in the lumen sound; disappearance or weakening of the spent response; phase change in the loss of blood spectrum; disappearance or weakening of the blood flow in the distal limb by squeezing.

### Statistical analysis

Descriptive statistics for continuous variables and categorical variables were recorded as mean ± standard deviation. Categorical variables were compared using Pearson’s χ^2^ test. Continuous variables were compared using Student’s *t*-test or Mann-Whitney rank sum test according to the normality of their distribution. Risk factors for LEDVT and D-dimer increase were studied by univariate and multivariate logistic regression analysis. A Student’s *t*-test was used to compare the D-dimer value before and after PICC insertion. Risk assessments were presented as odds ratios (ORs) with 95 % confidence intervals (CIs). The receiver operating characteristic (ROC) curve and the area under the curve (AUC) were used to evaluate the diagnostic capability and accuracy of D-dimer. All statistical analyses were conducted with SPSS (Version 18; SPSS, Central South University, Hunan, China). A *P*-value < 0.05 (two-tailed) was considered significant.

## Result

### PICC insertion increases the risk of LEDVT

Among the 3452 patients, 270 patients (7.82 %, including 43 patients inserted with PICCs) were diagnosed with LEDVT by ultrasound. Among the 270 patients, 67 cases were diagnosed by CDU due to thrombotic symptoms during the short follow up, and 203 cases were asymptomatic LEDVTs diagnosed by vascular ultrasound. Among the 3182 patients (92.18 %) without LEDVT, 61 patients were inserted with PICCs. LEDVT occurred in 43 of 104 patients inserted with PICCs 6.8 days (an average value) after catheterization. The median time from index to LEDVT was five days. Univariate logistic regression analysis showed that malignancy, PICC insertion, recent surgery, body mass index (BMI ≥ 25), unconsciousness, critical illness condition and diseases including ischemic stroke and Parkinson’s disease were associated with the increased risk of LEDVT (Table 2). PICC insertion increased the risk of LEDVT by 9 times (Table 2, OR = 9.692 [95 % CI: 6.414–14.646], *P* = 0.000). After adjustment of the risk factors, multivariate logistic regression analysis showed that PICC insertion increased the risk of LEDVT by 4 times (Table 2, OR = 4.268 [95 % CI: 2.501–7.282], *P* = 0.000).

**Table 2:** Logistic regression analysis of LEDVT-related factors

Factors	LEDVT	NO LEDVT	Univariate		Multivariate	
	*n* = 270	*n* = 3182	OR (95 % CI)	*P*	OR (95 % CI)	*P*
PICC	43	61	9.692 (6.414–14.646)	0.000	4.268 (2.501–7.282)	0.000
Age (year, mean ± standard deviation)	61.27 ± 12.63	61.57 ± 13.71	0.998 (0.989–1.007)	0.724	1.005 (0.995–1.015)	0.325
Male	156	1941	0.875 (0.680–1.125)	0.298	1.036 (0.788–1.360)	0.802
Malignant tumor	48	79	8.493 (5.787–12.464)	0.000	1.856 (1.038–3.321)	0.037
Recent surgery	80	160	7.593 (5.857–10.797)	0.000	4.056 (2.503–6.571)	0.000
Cerebral hemorrhage	26	210	1.508 (0.983–2.313)	0.060	0.699 (0.421–1.161)	0.167
Ischemic stroke	74	1539	0.403 (0.306–0.531)	0.000	0.562 (0.411–0.768)	0.000
Parkinson’s disease	8	222	0.407 (0.199–0.834)	0.014	0.511 (0.245–1.067)	0.074
Infection	26	311	0.984 (0.646–1.499)	0.939	1.597 (1.016–2.508)	0.042
BMI ≥ 25	29	209	1.712 (1.136–2.579)	0.010	1.325 (0.838–2.096)	0.229
Unconsciousness	78	549	1.948 (1.474–2.575)	0.000	1.007 (0.691–1.467)	0.972
Critical illness	57	117	7.010 (4.961–9.906)	0.000	5.179 (3.390–7.911)	0.000

Note: *PICC* refers to peripherally inserted central venous catheter; *BMI* refers to body mass index; malignant tumor refers to cases treated within the prior six months; recent surgery refers to neurosurgery (over two hours) performed within six months; critical illness refers to the circumstance where patients have a high risk of disease variation or death

### PICC insertion is associated with the increased risk of D-dimer

Patients with a high-risk Caprini score were inserted with PICCs. Among the 3452 patients, 1360 patients underwent D-dimer test within seven days after PICC insertion. Among the 1360 patients, 597 patients (43.90 %, including 80 patients inserted with PICCs) had a D-dimer value > 0.5 mg/L, and 763 patients (56.10 %, including 21 patients inserted with PICCs) had a D-dimer value ≤ 0.5 mg/L. Univariate logistic regression analysis showed that age, male sex, malignancy, PICC insertion, recent surgery, unconsciousness and diseases including cerebral hemorrhage, ischemic stroke, critical illness condition, Parkinson’s disease and infection were associated with the increased risk of D-dimer value (Table 3). PICC insertion promoted the increase of D-dimer value by 5 times (Table 3, OR = 5.467 [95 % CI: 3.338–8.956], *P* = 0.000). After adjustment of the risk factors, multivariate logistic regression analysis showed that PICC insertion promoted the increase of D-dimer value more than threefold (Table 3, OR = 3.354 [95 % CI: 1.773–6.346], *P* = 0.000).

**Table 3:** Logistic regression analysis of D-dimer-related factors

Factors	D-dimer (> 0.5 mg/L)	D-dimer (≤ 0.5 mg/L)	Univariate		Multivariate	
	*n* = 597	*s* = 763	OR (95 % CI)	*P*	OR (95 % CI)	*P*
PICC	80	21	5.467 (3.338–8.956)	0.000	3.354 (1.733–6.346)	0.000
Age (year, mean ± standard deviation)	61.91 ± 15.50	60.17 ± 14.46	1.008 (1.001–1.015)	0.033	1.014 (1.006–1.022)	0.001
Male	339	486	0.749 (0.602–0.932)	0.010	0.852 (0.670–1.082)	0.189
Malignant tumor	85	38	3.167 (2.126–4.720)	0.000	0.785 (0.430–1.435)	0.432
Recent surgery	158	64	3.931 (2.872–5.381)	0.000	4.944 (3.015–8.107)	0.000
Cerebral hemorrhage	58	41	1.895 (1.251–2.870)	0.003	1.245 (0.772–2.009)	0.369
Ischemic stroke	192	330	0.622 (0.497–0.778)	0.000	0.691 (0.531–0.901)	0.006
Parkinson’s disease	19	64	0.359 (0.213–0.606)	0.000	0.461 (0.266-0.800)	0.006
Infection	121	85	2.028 (1.500–2.740)	0.000	3.078 (2.218–4.271)	0.000
BMI ≥ 25	42	57	0.937 (0.620–1.148)	0.759	0.641 (0.400-1.026)	0.064
Unconsciousness	126	127	1.340 (1.019–1.762)	0.036	0.828 (0.579–1.184)	0.301
Critical illness	78	38	2.867 (1.915–4.294)	0.000	2.844 (1.754–4.613)	0.000

Note: *PICC* refers to peripherally inserted central venous catheter; *BMI *refers to body mass index; malignant tumor refers to cases treated within the prior six months; recent surgery refers to neurosurgery (over two hours) performed within six months; critical illness refers to the circumstance where patients have a high risk of disease variation or death

### D-dimer value before and after PICC insertion

Ninety-two patients underwent D-dimer tests within seven days before and after catheterization. During the testing period, none of the patients were pregnant, took oral contraceptive, underwent major surgery, or had new tumor, heart failure, infection or trauma/fracture. The D-dimer value was significantly increased within seven days after the PICC insertion (Table 4, *P* < 0.05).

**Table 4:** D-dimer changes before and after PICC insertion

	N	D-dimer (mg/L)	*t*	*P*
Before PICC insertion	92	0.86 ± 0.84	-7.07	0.000
After PICC insertion	92	1.78 ± 1.45		

Note: *PICC* refers to peripherally inserted central venous catheter

### Analysis of the predictive value of D-dimer for LEDVT

**Fig. 1:**
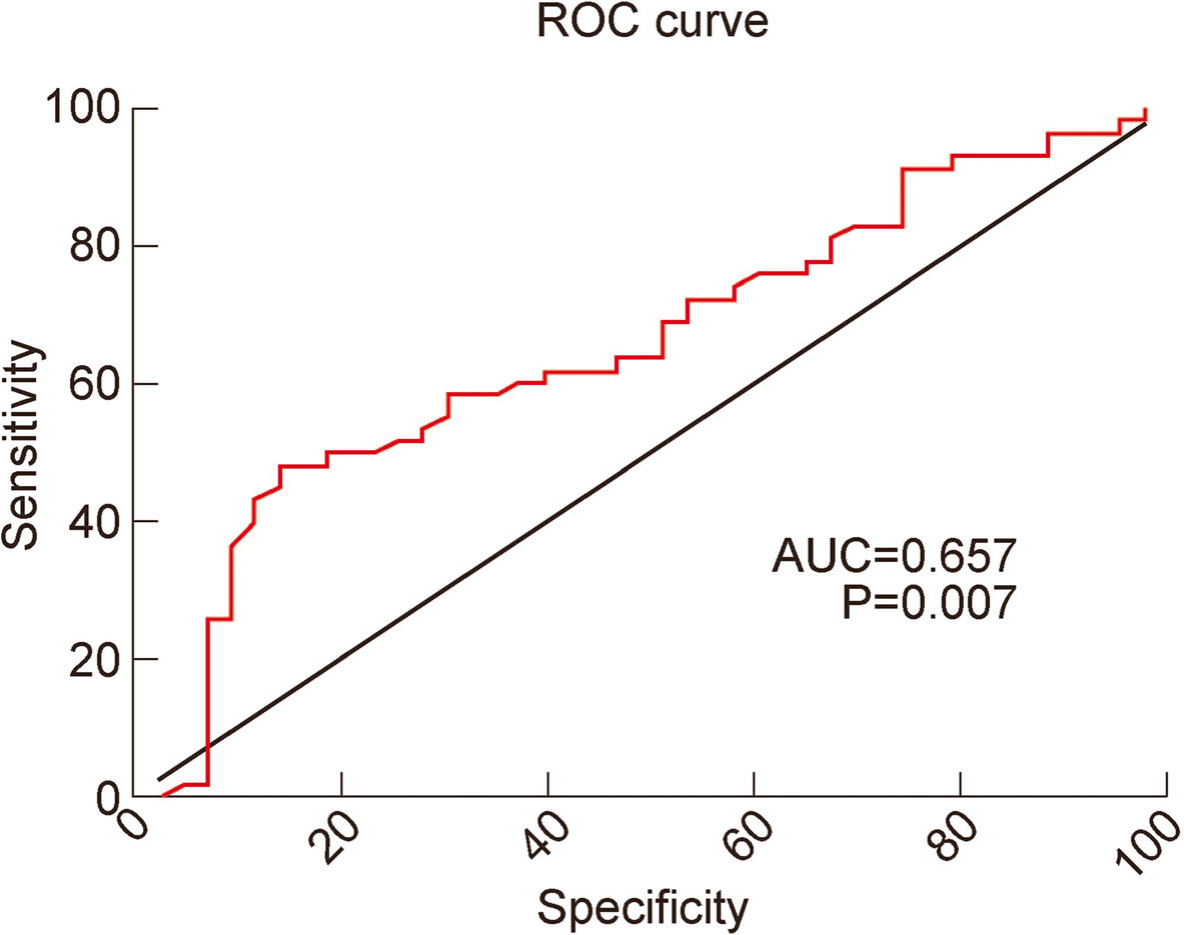
Area under ROC curve of D-dimer for diagnosis of LEDVT in patients inserted with PICCs. PICC, peripherally inserted central venous catheter; LEDVT, lower extremity deep venous thrombosis; ROC, receiver operating characteristic curve

ROC curves were plotted to evaluate the diagnostic sensitivity and specificity of D-dimer for LEDVT in patients with or without PICCs. The AUC of D-dimer in patients inserted with PICCs was 0.657 (Table 5; Fig. 1 and 95 % CI: 0.549–0.765, *P* < 0.05). The optimal critical value of D-dimer in patients inserted with PICCs was 0.675 mg/L, and the sensitivity, specificity, positive predictive value and negative predictive value of D-dimer were 48.28 %, 86.05 %, 55.22 and 82.35 %, respectively (Table 5).

**Table 5:** Analysis of the predictive value of D-dimer for LEDVT

	Optimal critical value	Sensitivity	Specificity	Positive predictive value	Negative predictive value	Area under ROC curve	95 % confidence interval
The level of D-dimer in patients with PICC	0.675	48.28 %	86.05 %	55.22 %	82.35 %	0.657	0.549–0.765
The level of D-dimer in patients without PICC	0.665	72.01 %	75.89 %	36.82 %	93.25 %	0.800	0.769–0.830

Note: *PICC* refers to peripherally inserted central venous catheter; *LEDVT* lower extremity deep venous thrombosis; *ROC* receiver operating characteristic curve

**Fig. 2:**
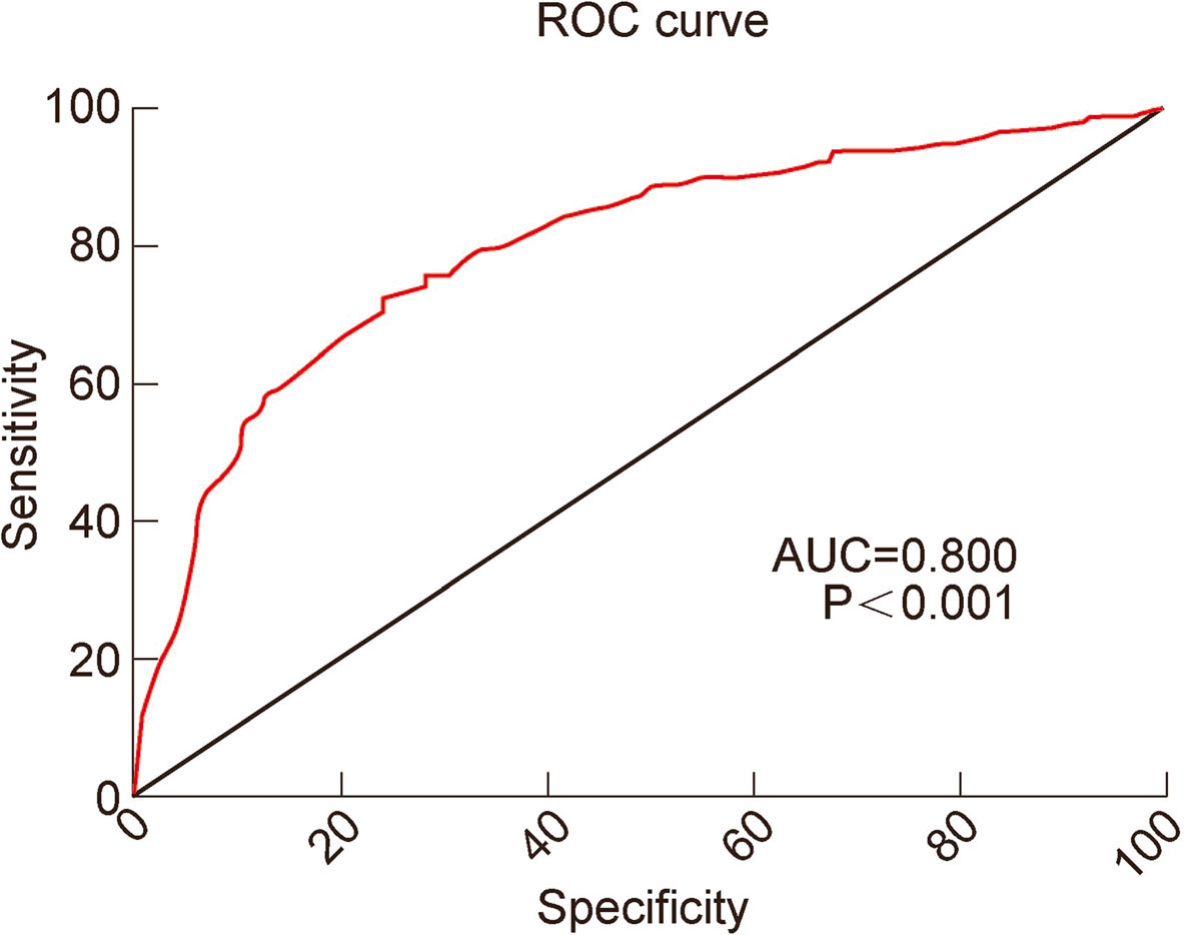
Area under ROC curve of D-dimer for diagnosis of LEDVT in patients without PICC. PICC, peripherally inserted central venous catheter; LEDVT, lower extremity deep venous thrombosis; ROC, receiver operating characteristic curve

The AUC of D-dimer in patients without PICC was 0.800 (Table 5; Fig. 2 and 95 % CI: 0.769–0.830, *P* < 0.05). The optimal critical value of D-dimer in patients without PICC was 0.665 mg/L, and the sensitivity, specificity, positive predictive value and negative predictive value of D-dimer were 72.01 %, 75.89 %, 36.82 and 93.25 %, respectively (Table 5).

The results show that the sensitivity, specificity, and positive predictive value of D-dimer are not high. Generally, the negative predictive value of D-dimer is used to exclude thrombosis. The negative predictive value of D-dimer in patients inserted with PICCs (82.35 %) is lower than in patients without PICC, suggesting D-dimer as an inappropriate predictor for thrombosis in patients inserted with PICCs.

## Discussion

Venous thrombosis is a common vascular disorder in the general population. The annual incidence of venous thromboembolism in people of European descent is estimated to be 104 to 183 per 100,000 people [29]. DVT more often happens in lower extremities than in upper extremities. In USA, LEDVT has an annual incidence in about 187,000 individuals and is considered as the third common vascular disease following myocardial infarction and stroke [30]. Registro Informatizado de Enfermedad Trombo Embólica (RIETE) enrolled 37,366 patients with DVT, among which 35,094 (93.9 %) cases were LEDVT and the other 2272 (6.1 %) were UEDVT [31]. A recent retrospective cohort study of 83 patients hospitalized for DVT showed that 72 cases were LEDVT and 11 cases were UEDVT [32]. Foreign bodies in the vascular system are the most important independent risk factors of DVT [33–35]. A study of the general population showed that LEDVT occurred in 372 of 3790 patients inserted with PICCs during hospitalization [3]. In this study, 43 of the 270 LEDVT patients were inserted with PICCs. Consistently, we found that PICC insertion increased the risk of LEDVT by 4 times after risk adjustment. LEDVT occurred in 43 of 104 patients inserted with PICCs 6.8 days (an average value) after catheterization. The median time from index to LEDVT was five days, which is similar to the time for UEDVT. PICC-related UEDVT occurs within 12 days after catheterization, and the median time for UEDVT is 8 days [36]. In addition to the PICC insertion, factors including malignancy, recent surgery, infection, BMI ≥ 25 and unconsciousness were also associated with the increased risk of LEDVT.

The concentration of D-dimer is considered a gold biochemical standard for assessing both coagulation activation and fibrin digestion [37] and for ruling out venous thrombosis in patients with low-median clinical probability [38–40]. Other conditions such as infection, cancer, chronic inflammation, aging, pregnancy, recent surgery, and trauma can also increase the concentration of D-dimer by accelerating fibrin production or breakdown [41]. A retrospective study of 1647 patients showed that the most common cause of positive D-dimer was infection, followed by venous thrombosis, syncope, heart failure, trauma, and cancer [42]. After excluding these factors, we investigated the D-dimer concentration within seven days before PICC catheterization. Evidence showed that the risk of PICC line-related DVT was increased in the first two weeks after PICC insertion [15]. We found that the concentration of D-dimer within seven days after PICC insertion was higher than that before PICC insertion, indicating a PICC-induced increase in D-dimer. Moreover, recent surgery, infection, and cerebral hemorrhage were also associated with the increase in D-dimer. D-dimer cannot be used to exclude venous thrombosis in patients inserted with PICCs.

Thrombosis is a natural process activated by internal and external pathways which stimulate series of coagulation in the body and eventually lead to fibrin rich thrombus. Endothelial injury is a coagulation inciting event. Fibrinolysis and destruction of the blood clot are induced following coagulation to maintain homeostasis. DVT occurs when the coagulation process is not restrained or the mechanism of decomposing blood clots is overloaded [43]. A PICC occupies nearly half of the inner diameter of a vein, which causes a slowing of the local blood flow. The stasis of blood flow can induce micro venous thrombosis and activate the coagulation system in the process of reflux, leading to a larger range of DVT [43]. We suspect that PICC insertion is a risk factor of LEDVT and results in a general increase in D-dimer concentration. By the joint action of other risk factors, DVT can spread beyond the PICC vascular bed.

This study provides novel insights for the clinical evaluation of LEDVT. A previous systematic search of the literature for PICC-related DVT summarized the risk factors, symptoms, diagnosis, management and prevention of this event [44]. However, how PICC insertion is related to DVT is barely known. In this study, the concentration of D-dimer was first used to explain the possible relationship between PICC inserted in upper extremities and LEDVT. A PICC inserted in upper extremities increases the risk of LEDVT, which inspires clinicians to consider the complications of PICC insertion. For patients with a high risk of thrombosis, it is necessary for a specialized nursing team to weigh the side-effect and benefit of PICC or to consider an alternative vascular access. Moreover, clinicians are suggested to use a prophylactic anticoagulant regimen in patients at high risk of thrombosis who need PICC insertion. This study suggested a connection between PICC insertion and subsequent LEDVT and another possibility is that the PICC insertion may be a marker of a sicker and higher risk patient. In this regards, we have added the factor critical illness and main risk factors for LEDVT for logistic regression analysis and the results supported this possibility.

Despite the significance, limitations exist in this study. Firstly, the data of asymptomatic UEDVT was not collected to determine whether PICC insertion conferred a greater risk of DVT in upper extremities than in lower extremities. Secondly, this study was limited to the neurology department.

## Conclusions

PICC insertion increases the value of D-dimer and is an important risk factor of LEDVT. The predictive value of D-dimer in patients inserted with PICCs is lower than in patients without PICC. D-dimer is unsuitable for routine examination to exclude LEDVT in patients inserted with PICCs.

## Data Availability

The datasets used and/or analyzed during the current study are available from the corresponding author on reasonable request.

## Abbreviations

PICC: Peripherally Inserted Central venous Catheter
LEDVT: Lower extremity deep venous thrombosis
UEDVT: Upper extremity deep venous thrombosis
